# Bacillus Calmette-Guérin (BCG) Brazilian Backstage in Bladder Cancer

**DOI:** 10.1590/S1677-5538.IBJU.2021.02.02

**Published:** 2021-02-03

**Affiliations:** 1 Faculdade de Medicina do ABC-FMABC Divisão de Urologia Santo André SP Brasil Divisão de Urologia, Faculdade de Medicina do ABC-FMABC, Santo André, SP, Brasil.; 2 Hospital Israelita Albert Einstein São Paulo SP Brasil Hospital Israelita Albert Einstein, São Paulo, SP, Brasil.; 3 Universidade Estadual de Campinas - Unicamp Campinas SP Brasil UroScience, Universidade Estadual de Campinas - Unicamp, Campinas, SP, Brasil.; 4 Pontifícia Universidade Católica de Campinas - PUC-Campinas Campinas SP Brasil Pontifícia Universidade Católica de Campinas - PUC-Campinas, Campinas, SP, Brasil.

## COMMENT

Bacillus Calmette-Guérin (BCG) is a live attenuated vaccine derived from Mycobacterium bovis initially isolated in 1902 from a tuberculous cow (1). This isolate was cultured for 13 years until it lost its virulence and was developed into a vaccine by Albert Calmette and Camille Guerin in 1921 at the Pasteur Institute in Lille, France. It is the only available vaccine to fight tuberculosis and other mycobacterial infections as leprosy and Buruli ulcer disease (2, 3).

All the BCG substrains currently in use derive from the original strain produced, distributed to several countries. Currently, the main substrains are the Brazilian (Moreau/Rio de Janeiro), Danish (Copenhagen - 1331), Japanese (Tokyo - 172-1), Russian (Moscow - 368), and Bulgarian (Sofia - SL222). The World Health Organization (WHO) has recommended BCG vaccination since 1948 and recognized the Brazilian Moreau-RJ as a reference strain in 2012, along with the Russian, Danish, and Japanese strains (3, 4).

During the 1970s, the Canadian urologist Alvaro Morales have studied BCG for treating non-muscle invasive bladder cancer (NMIBC) (5). Since then, intravesical BCG is still the most effective treatment, along with transurethral resection, to avoid NMIBC recurrence. According to all international guidelines, it is recommended as first-line therapy for NMIBC, as it reduces to almost half the recurrence rates (6–8).

In addition to proven efficacy as a vaccine on genitourinary oncology, BCG is still the target for molecular and cellular research since the mechanisms activated by vaccination are complex, involving at least two responses: T cells antigen-specific and memory (classic adaptive immunity), and “trained” immunity (epigenetic reprogramming) mediated by innate immune cells (9, 10). This reprogramming occurs at the histone methylation level, inducing persistent conformational changes of chromatin in a mononuclear cell, exhibiting a long-lasting phenotype after vaccination with the ability to mediate specific and nonspecific protection against other infections, leading to a robust and faster immune response compared to adaptive immunity (9–12).

In such background, BCG is an alternative to battle agents of the most diverse natures, since the immune system is continuously tackled, especially within 2020 current context in which BCG has been challenged in clinical trials against COVID-19 as potential preventive and even therapeutic strategy to boost the immune system (13–15). In the placebo controlled, randomized, double-blind phase III ACTIVATE study, published on August 31, 2020 in the important journal “CELL”, BCG vaccination in elderly patients proved to be safe with no difference in the frequency of adverse effects, and with an 80% decrease in respiratory infections (risk ratio 0.21, p = 0.013) (16).

While onco-BCG is more profitable for manufacturers than their tuberculosis vaccine counterpart, the latter is a public health priority. From over thirty global manufacturers during the 1990s, in 2017, only 17 manufacturers worldwide kept producing BCG (17). In the last three years, some additional manufacturers have withdrawn production. In 2011, the Food and Drug Administration (FDA) shuttered a Sanofi Pasteur's manufacturing laboratory in Toronto for health issues and mold after a flood. It never reopened. In the same year, Institute Cantacuzino, in Romania, also decided to stop BCG production. In 2014, the Danish government decided to sell the BCG vaccine production business. In 2016, Merck also experienced manufacturing issues. In the USA, Merck is currently the sole supplier of BCG (18).

## The Brazilian backstage

The first BCG vaccine sample has arrived in Brazil in 1925, brought by the Uruguayan doctor Julio Elvio Moreau. That sample was delivered to Dr. Arlindo Raymundo de Assis in Rio de Janeiro and was called BCG Moreau (4). Given the difficulties in bringing laboratory supplies from Europe at that time, the vaccine was initially produced with the Viscondesa de Morais culture media's, which was not the original culture media recommended by the Pasteur Institute. These adaptations were responsible for mutations in the microorganisms, and it is noteworthy the lower rate of adverse effects of this strain (19).

Since the 1930s, the Moreau strain has been maintained by the Fundação Ataulpho de Paiva (FAP), a nonprofit foundation in Rio de Janeiro, Brazil and after the 1970s the lyophilization process has been adopted. In the 1980s, the Instituto Butantan, a public research institution in São Paulo, also started to produce BCG from the Moreau-RJ strain. In 2003, the bladder cancer-targeted “onco-BCG” production also started at Instituto Butantan; however, the production was adapted at antique production laboratories that did not respect the WHO standards. After only four years, in 2007, the production of BCG ceased in São Paulo.

Supply availability has been the main challenge with BCG production in Brazil as in the rest of the world, and there are several reasons for that. While vaccine development technologies have widely progressed, BCG manufacturing has remained mostly unchanged since the 1920s. Surprisingly there are no reports of significant contamination problems during the 86 years of BCG production in Brazil.

Since 1992 the WHO has issued and updated the Good Manufacturing Practices for Biological Products (GMP) recommendations (20). It is an important scientific and advisory document aiming to guide regulatory agencies and manufacturers. Nevertheless, there are some difficulties for BCG production since filtration processes cannot sterilize the BCG vaccine. For these axenic products, processing should be conducted aseptically to minimize the introduction of contaminants. For that reason, robust environmental controls and monitoring are essential to avoid contamination. In contrast to these GMP issues, suppliers and manufacturers’ decisions are associated with this low-price vaccine. While the relatively low cost of BCG production makes it widely affordable, they are also associated with reduced incentives for manufacturers to enhance and invest in the production processes.

WHO regional offices have tracked BCG shortages for children immunization during the last fifteen years. BCG shortages have been a problem worldwide since then, and developing countries have been even more affected (17). If supply problems have been common for the widely applied, largely demanded, BCG vaccine, that uses a low dose (0.05 ml to 0.1 ml) per vial, it is even a bigger problem for onco-BCG that relies on higher doses (80mg). Even though there is no precise tracking for the onco-BCG shortage worldwide, supply problems have been frequent in Brazil during the last four years.

In Brazil, FAP is the sole supplier. In 2016, FAP started to experience manufacturing issues with the Brazilian Health Regulatory Agency (ANVISA), as their production did not attend GMP recommendations. Non-conformities included four critic, 25 major, and 14 minor problems (21). According to FAP representatives, GMP rules are extremely difficult to be followed. Currently, GMP does not tolerate any particles in the final product. The lyophilizers must be altered for expensive ones with self-cleaning technologies. Air conditioning structures must be rebuilt; the water supply system must be reformatted. BCG is an artisanal four-month process of a potato cultivated mycobacteria that result in a low-value product. In such a context, it is not easy to implement technological processes. BCG passes through several Erlenmeyers during the cultivation process. After a 21-day cultivation period, the bacterial suspension must be repeatedly diluted for the established number of doses. The first product is evaluated according to its colony formation units (CFU) and sterility. The product must be axenic, and at least six animals must be tested for 42 days.

At FAP, the solution would be a completely automated process and rebuilding new BCG manufacturing industries. Since FAP's old manufacturing unit, in São Cristóvão could not attend GMP requirements, a new unit has been under construction, in Xerém, Duque de Caxias, during the last decades. The construction of this new FAP's industry in Xerém, RJ, has been largely awaited. This unit has been under construction since 1989, costing several millions of dollars but still not working. This unit aims to respect GMP's strict rules. Furthermore, currently, there is a technology transfer agreement between FAP, in Brazil, and a Spanish institution, with a new manufacturing center being developed in Madrid to supply the European BCG market. As in many countries, in Brazil, only one strain is registered. The Moreau-RJ strain is only produced by the FAP. Another Moreau strain is produced in Poland by Biomed Lublin.

At the same time of BCG shortage, bladder cancer incidence has been increasing in Brazil in the last decades, with 10.640 cases expected for each year of the 2020/21/22 triennium, while in 2018-2019, the estimate was 9.480 cases per yearc(22). Along with the populational aging process, health care access improvement, and an increase in BC related hospitalizations make it reasonable to expect an increase of BCG requirements in the next years (22, 23).

When BCG is not available, intravesical chemotherapies or immunotherapeutics, as pembrolizumab (24), can be more rentable and less effective treatment strategies (25), featuring as another negative incentive to BCG production. While BCG prices range between US$ 60-70 each vial (2×10^6^ CFU/40mg), chemotherapies as mitomycin had their prices almost doubled recently, costing around US$ 1,000.00/ vial (18) and immunotherapeutics cost around US$ 6,000.00/vial (25).

During the last years, while the FAP production was halted, onco-BCG only became available to wealthiest Brazilians. During the frequent periods of BCG shortage in the last four years, the only alternative is to directly import, at prices as high as US$ 5,000.00/12 vials (SWOG induction protocol) (26), instead of US$ 720.00. Additionally, the government does not acquire the onco-BCG directly from FAP anymore, and each hospital has to buy its supply through independent distributors. Right now, most patients are not receiving BCG treatment (or any treatment), and many patients are progressing to invasive disease and dying of BC much more than they should.

Right now, FAP is authorized by ANVISA to sell onco-BCG vials again. However, a new audit will probably occur within the next months, with a new possible interruption of onco-BCG distribution, as GMP requirements will probably not be attended. The costs, including financial expenses, human lives, and the lack of adequate treatment, are not measured. Nevertheless, there is no doubt that treating patients with more advanced BC is significantly costlier, associated with more suffering and deaths. BCG production is a problem that has to be seriously addressed to come to a definitive solution in Brazil and all over the globe.

## References

[1] 1. Luca S, Mihaescu T. History of BCG Vaccine. Maedica (Buchar). 2013;8:53-8. PMC3749764 24023600

[2] 2. Fine PEM, Carneiro IM, Milstein JB, Clements CJ. Issues relating to the use of BCG in immunization programmes - A discussion document. WHO. 1999; 1-45. [Internet]. Available at. <https://apps.who.int/iris/handle/10665/66120>

[3] 3. Fine PEM, Carneiro IM, Milstein JB, Clements CJ. BCG vaccines: WHO position paper - February 2018. Wkly Epidemiol Rec. 2018;93:73-96.

[4] 4. Benévolo-de-Andrade TC, Monteiro-Maia R, Cosgrove C, Castello-Branco LR. BCG Moreau Rio de Janeiro: an oral vaccine against tuberculosis--review. Mem Inst Oswaldo Cruz. 2005;100:459-65. 10.1590/s0074-02762005000500002 16184220

[5] 5. Morales A, Eidinger D, Bruce AW. Intracavitary Bacillus Calmette-Guerin in the treatment of superficial bladder tumors. J Urol. 1976;116:180-3. 10.1016/s0022-5347(17)58737-6 820877

[6] 6. Babjuk M, Burger M, Compérat EM, Gontero P, Mostafid AH, Palou J, et al. European Association of Urology Guidelines on Nonmuscle-invasive Bladder Cancer (TaT1 and Carcinoma In Situ) - 2019 Update. Eur Urol. 2019;76:639-57. 10.1016/j.eururo.2019.08.016 31443960

[7] 7. Reis LO, Moro JC, Ribeiro LF, Voris BR, Sadi MV. Are we following the guidelines on non-muscle invasive bladder cancer? Int Braz J Urol. 2016;42:22-8. 10.1590/S1677-5538.IBJU.2015.0122 PMC4811222 27136464

[8] 8. Herr HW, Wartinger DD, Fair WR, Oettgen HF. Bacillus Calmette-Guerin therapy for superficial bladder cancer: a 10-year followup. J Urol. 1992;147:1020-3. 10.1016/s0022-5347(17)37452-9 1552578

[9] 9. Kleinnijenhuis J, Quintin J, Preijers F, Joosten LA, Ifrim DC, Saeed S, et al. Bacille Calmette-Guerin induces NOD2-dependent nonspecific protection from reinfection via epigenetic reprogramming of monocytes. Proc Natl Acad Sci USA. 2012;109:17537-42. 10.1073/pnas.1202870109 PMC3491454 22988082

[10] 10. Arts RJW, Carvalho A, La Rocca C, Palma C, Rodrigues F, Silvestre R, et al. Immunometabolic Pathways in BCG-Induced Trained Immunity. Cell Rep. 2016;17:2562-71. 10.1016/j.celrep.2016.11.011 PMC5177620 27926861

[11] 11. Moorlag SJCFM, Arts RJW, van Crevel R, Netea MG. Non-specific effects of BCG vaccine on viral infections. Clin Microbiol Infect. 2019;25:1473-8. 10.1016/j.cmi.2019.04.020 31055165

[12] 12. Khader SA, Divangahi M, Hanekom W, Hill PC, Maeurer M, Makar KW, et al. Targeting innate immunity for tuberculosis vaccination. J Clin Invest. 2019;129:3482-91. 10.1172/JCI128877 PMC6715374 31478909

[13] 13. Reis LO, Ferrari KL. COVID-19: BCG As Therapeutic Vaccine, Transmission Limitation, and Immunoglobulin Enhancement (BATTLE). Clinicaltrials.gov 2020. [Internet]. Available at. <https://clinicaltrials.gov/ct2/show/NCT04369794>

[14] 14. Gursel M, Gursel I. Is global BCG vaccination-induced trained immunity relevant to the progression of SARS-CoV-2 pandemic? Allergy. 2020;75:1815-9. 10.1111/all.14345 PMC7267226 32339299

[15] 15. O’Neill LAJ, Netea MG. BCG-induced trained immunity: can it offer protection against COVID-19? Nat Rev Immunol. 2020;20:335-7. 10.1038/s41577-020-0337-y PMC7212510 32393823

[16] 16. Giamarellos-Bourboulis EJ, Tsilika M, Moorlag S, Antonakos N, Kotsaki A, Domínguez-Andrés J, et al. Activate: Randomized Clinical Trial of BCG Vaccination against Infection in the Elderly. Cell. 2020;183:315-23. 10.1016/j.cell.2020.08.051 PMC7462457 32941801

[17] 17. Cernuschi T, Malvolti S, Nickels E, Friede M. Bacillus Calmette-Guérin (BCG) vaccine: A global assessment of demand and supply balance. Vaccine. 2018;36:498-506. 10.1016/j.vaccine.2017.12.010 PMC5777639 29254839

[18] 18. Keshavan M. Supplies of a bladder cancer drug are dwindling, as patients scramble. 2019 [Internet]. Available at. <https://www.statnews.com/2019/02/20/supplies-bladder-cancer-drug-bcg-dwindling/>

[19] 19. Chen JM, Islam ST, Ren H, Liu J. Differential productions of lipid virulence factors among BCG vaccine strains and implications on BCG safety. Vaccine. 2007;25:8114-22. 10.1016/j.vaccine.2007.09.041 17954004

[20] 20. Ahmed MMF, Kopp S, Lei D. World Health Organization - Good manufacturing practices for pharmaceutical - Annex 2. WHO Expert Comm. Specif. Pharm. Prep. WHO Techni, 2016:77-136. [Internet]. Available at. <https://www.who.int/medicines/areas/quality_safety/quality_assurance/TRS986annex2.pdf>

[21] 21. [No Authors]. Fabricação de vacinas BCG e Imuno BCG é suspensa. Ascom/ANVISA. December, 12 [Intenet]. Available at. <https://www.gov.br/anvisa/pt-br/assuntos/noticias-anvisa/2018/fabricacao-de-vacinas-bcg-e-imuno-bcg-e-suspensa>

[22] 22. Timoteo F, Korkes F, Baccaglini W, Glina S. Bladder cancer trends and mortality in the brazilian public health system. Int Braz J Urol. 2020;46:224-33. 10.1590/S1677-5538.IBJU.2019.0198 PMC7025845 32022511

[23] 23. Schilithz A, Lima FCS, Oliveira JFP, Santos MO, Rebelo MS. Estimativa da Incidência e Mortalidade por Câncer no Brasil - 2020. Instituto Nacional do Cancer José Alencar Gomes da Silva. INCA. 2019. [Internet]. Available at. <https://www.inca.gov.br/sites/ufu.sti.inca.local/files/media/document/estimativa-2020-incidencia-de-cancer-no-brasil.pdf>

[24] 24. [No Authors]. FDA approves pembrolizumab for BCG-unresponsive, high-risk non-muscle invasive bladder cancer. FDA. 2020. [Internet]. Available at. <https://www.fda.gov/drugs/resources-information-approved-drugs/fda-approves-pembrolizumab-bcg-unresponsive-high-risk-non-muscle-invasive-bladder-cancer>.

[25] 25. Korkes F, Maluf F. Increasing costs from bladder cancer in the Brazilian Health System: the role of establishing public health policies. Int Braz J Urol. 2021;47:443-7. 10.1590/S1677-5538.IBJU.2020.0658 PMC7857771 33284548

[26] 26. Lamm DL, Blumenstein BA, Crissman JD, Montie JE, Gottesman JE, Lowe BA, et al. Maintenance bacillus Calmette-Guerin immunotherapy for recurrent TA, T1 and carcinoma in situ transitional cell carcinoma of the bladder: a randomized Southwest Oncology Group Study. J Urol. 2000;163:1124-9. 10737480

